# Sibling cell size matters

**DOI:** 10.7554/eLife.24038

**Published:** 2017-01-13

**Authors:** Clemens Cabernard

**Affiliations:** 1 Department of Biology, University of Washington, Seattle, United Statesccabern@uw.edu

**Keywords:** stem cells, centrosome, asymmetric cell division, *D. melanogaster*

## Abstract

A motor protein called Klp10A ensures that germline stem cells in male fruit flies divide to produce two sibling cells that are equal in size.

**Related research article** Chen C, Inaba M, Venkei ZG, Yamashita YM. 2016. Klp10A, a stem cell centrosome-enriched kinesin, balances asymmetries in *Drosophila* male germline stem cell division. *eLife* **5**:e20977. doi: 10.7554/eLife.20977

Cell division is a highly regulated and tightly choreographed process. It ensures that the DNA, organelles and other components in a cell are correctly distributed between the two "sibling" cells that are produced during the cell division process. In most cases, the dividing cell ensures that the sibling cells are near identical in size. However, many types of cells, including baker’s yeast and cells in animal ovaries, produce sibling cells of different sizes. How and why dividing cells regulate the sizes of the sibling cells are unresolved questions in cell biology (Roubinet and Cabernard, 2014). Now, in eLife, Cuie Chen, Mayu Inaba, Zsolt Venkei and Yukiko Yamashita of the University of Michigan report a new mechanism that dividing cells use to ensure that both sibling cells are equal in size (Chen et al., 2016).

In animal and other eukaryotic cells, DNA is packaged into structures called chromosomes. During cell division, the chromosomes in a cell are divided into two groups by a structure called the spindle apparatus. In animal cells two organelles called centrosomes help to build the spindle apparatus (Nigg and Raff, 2009). It is important that the spindle apparatus is assembled correctly because asymmetric spindles could exert uneven spindle forces and may result in the sibling cells having incorrect numbers of chromosomes.

In the testes of male fruit flies, germline stem cells divide to produce one new germline stem cell and one gonialblast (which will go on to produce sperm cells) that are equal in size. Chen et al. found that the centrosomes of germline stem cells contain high levels of a motor protein called Klp10A. Decreasing the amount of Klp10A in these cells causes one of the centrosomes – presumably the older "mother" centrosome – to become much longer than normal. This, in turn, leads to the formation of asymmetric spindles and results in a new germline stem cell that is significantly larger than the gonialblast (Figure 1A,B). Despite the importance of centrosome activity for chromosome segregation, all of the chromosomes (except for the small fourth chromosome) segregate normally in Klp10A depleted germline stem cells.

**Figure 1.**
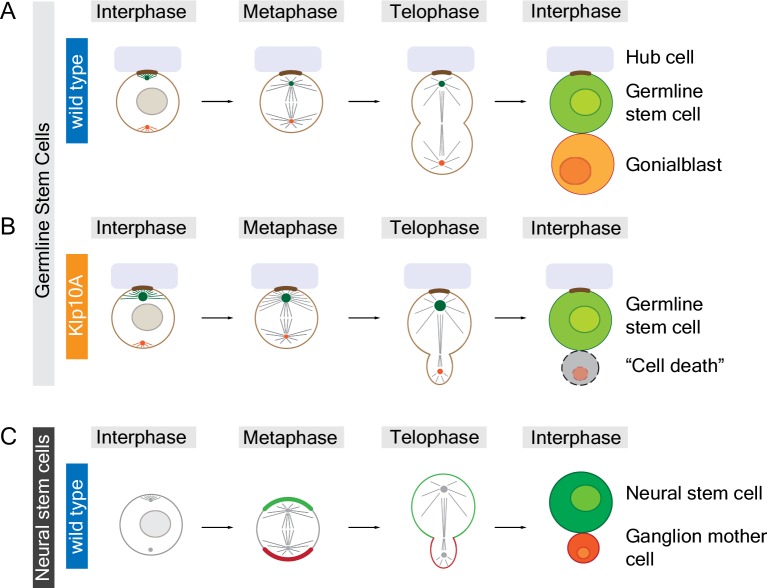
Animal cells can divide to produce sibling cells that are equal or unequal in size. Structures called centrosomes help to organize networks of microtubules (straight lines) inside cells. (**A**) Early in the cell cycle (during interphase; left column) the mother centrosome (green) in a fruit fly male germline stem cell is slightly more active than the other centrosome (red), which helps to anchor (brown) the stem cell to the hub cell (pale purple). Later in the cell cycle (during metaphase and telophase) both centrosomes are equally active, organizing the microtubules to form a symmetrical spindle apparatus across the center of the cell. When the cell divides it produces a new germline stem cell and a gonialblast that are equal in size. (**B**) The mother centrosome in a Klp10A depleted germline stem cell is more active than the other centrosome in interphase, metaphase and telophase, which leads to an asymmetric spindle and siblings of different size. The larger cell remains attached to the hub cell, adopting stem cell fate, whereas the smaller sibling often dies. (**C**) Neural stem cells in fruit flies are intrinsically polarized (green and red crescent, respectively) and also contain asymmetric centrosomes, which result in an asymmetric spindle at telophase. The larger cell is destined to become the neural stem cell whereas the smaller cell becomes a ganglion mother cell. In contrast to germline stem cells, it is the asymmetric localization of motor proteins in neural stem cells during anaphase (between metaphase and telophase; not shown) that is largely responsible for producing sibling cells that are unequal in size. Small circles in interphase cells represent cell nuclei; the chromosomes are not shown.

This imbalance in centrosome activity seems to be specific to male germline stem cells since Klp10A depleted cells that are destined to become sperm do not show this behavior. A possible explanation is that male germline stem cells – like other stem cells – segregate their centrosomes asymmetrically during cell division with the new stem cell always retaining the mother centrosome. It is also possible that this specificity is due to the fact that germline stem cells are attached to hub cells, which provide a niche environment for the stem cells.

Why do germline stem cells need to form sibling cells of equal size? Chen et al. addressed this question by using live cell imaging to follow the fates of sibling cells in Klp10A depleted testes. These experiments revealed that the smaller gonialblasts often die. This is unlikely to be due to the mis-segregation of the fourth chromosome (because it is not essential for cells to survive; Gelbart, 1974), but may be caused by differences in the segregation of organelles between the sibling cells. For example, the smaller gonialblasts inherit more mitochondria, but less Golgi, than the gonialblasts in normal testes.

The observations reported by Chen et al. agree with recent reports from teams led by Iain Cheeseman (Kiyomitsu and Cheeseman, 2013) and Patrick Meraldi (Tan et al., 2015). They showed that changes in spindle position or the location of the metaphase plate (where chromosomes line up before the cell divides) can induce asymmetric division of animal cells that, under normal conditions, always produce equally sized siblings. Similar to the small gonialblasts in fruit fly testes, the smaller siblings died or spent longer preparing for cell division. There is currently no molecular explanation for how differences in sibling cell size could affect cell fate, but it is possible that altered segregation of cell organelles, a cell size checkpoint or cell competition may be responsible.

Whether animal cells produce sibling cells that are equal or unequal in size seems to be tightly controlled during development. In contrast to germline stem cells, neural stem cells in fruit flies develop asymmetric spindles so that, when they divide, the new neural stem cell is larger than its sibling (Homem and Knoblich, 2012; Figure 1C). If neural stem cells are forced to divide symmetrically they produce two new neural stem cells of equal size (Cabernard and Doe, 2009). Similarly, some neural stem cells in the worm *Caenorhabditis elegans* also produce siblings of different sizes (Ou et al., 2010). In both of these examples, the difference in cell size seems to be primarily controlled through asymmetric localization of a motor protein called non-muscle myosin, which drives cell division, and not through inherent asymmetries in the spindle (Ou et al., 2010; Cabernard et al., 2010; Connell et al., 2011). Thus, nature has developed at least two independent mechanisms to ensure that sibling cells adopt the right size.

It is currently not clear how Klp10A regulates the size of centrosomes, or what molecular mechanisms regulate spindle asymmetry in germline stem cells and other systems. In the future it may be possible to develop tools that allow us to artificially change the relative sizes of sibling cells in order to investigate how this affects animal development.
